# Malpighian Tubules as Novel Targets for Mosquito Control

**DOI:** 10.3390/ijerph14020111

**Published:** 2017-01-24

**Authors:** Peter M. Piermarini, Carlos J. Esquivel, Jerod S. Denton

**Affiliations:** 1 Department of Entomology, Ohio Agricultural Research and Development Center, The Ohio State University, Wooster, OH 44691, USA; esquivelpalma.1@buckeyemail.osu.edu; 2 Departments of Anesthesiology and Pharmacology, Vanderbilt University Medical Center, Nashville, TN 37232, USA; jerod.s.denton@Vanderbilt.Edu

**Keywords:** Malpighian tubules, mosquito, Kir channels, insecticides, small molecule, homeostasis, detoxification

## Abstract

The Malpighian tubules and hindgut are the renal excretory tissues of mosquitoes; they are essential to maintaining hemolymph water and solute homeostasis. Moreover, they make important contributions to detoxifying metabolic wastes and xenobiotics in the hemolymph. We have focused on elucidating the molecular mechanisms of Malpighian tubule function in adult female mosquitoes and developing chemical tools as prototypes for next-generation mosquitocides that would act via a novel mechanism of action (i.e., renal failure). To date, we have targeted inward rectifier potassium (Kir) channels expressed in the Malpighian tubules of the yellow fever mosquito *Aedes aegypti* and malaria mosquito *Anopheles gambiae*. Inhibition of these channels with small molecules inhibits transepithelial K^+^ and fluid secretion in Malpighian tubules, leading to a disruption of hemolymph K^+^ and fluid homeostasis in adult female mosquitoes. In addition, we have used next-generation sequencing to characterize the transcriptome of Malpighian tubules in the Asian tiger mosquito *Aedes albopictus*, before and after blood meals, to reveal new molecular targets for potentially disrupting Malpighian tubule function. Within 24 h after a blood meal, the Malpighian tubules enhance the mRNA expression of genes encoding mechanisms involved with the detoxification of metabolic wastes produced during blood digestion (e.g., heme, NH_3_, reactive oxygen species). The development of chemical tools targeting these molecular mechanisms in Malpighian tubules may offer a promising avenue for the development of mosquitocides that are highly-selective against hematophagous females, which are the only life stage that transmits pathogens.

## 1. Introduction

Adult female mosquitoes are vectors of numerous pathogens that cause diseases of significance to global health. Notably, hundreds of millions of people are infected with mosquito-borne malaria and dengue fever each year, leading to over 500,000 deaths [1,2]. Moreover, chikungunya fever and Zika virus, which were historically limited to parts of Africa and Asia, have recently emerged into global threats with unprecedented autochthonous transmission by mosquitoes in the Western hemisphere [3,4]. Effective, low-cost therapeutics and/or vaccines for treating and preventing these diseases have either not been developed or are not widely-available. Thus, the primary—and only universal—means to limit the spread of mosquito-borne diseases is to implement control of the mosquito vector, especially when a previously neglected arbovirus is emerging (e.g., Zika).

Chemical control of mosquitoes with insecticides remains a cornerstone to integrated vector management, especially in regions of the world where biological and genetic approaches are cost prohibitive or not logistically feasible. Most insecticides used for mosquito control target the nervous system, such as pyrethroids, carbamates, organophosphates, and organochlorides. Although these neurotoxins are highly effective at killing mosquitoes, the overuse of a limited number of active compounds has exerted a strong, selective pressure for traits that make mosquitoes resistant to conventional insecticides (e.g., knockdown resistance mutations, elevated detoxification mechanisms) [5], analogous to the evolution of antibiotic resistance in pathogenic bacteria. Thus, the number of effective chemical tools available for mosquito control is diminishing, presenting an emerging challenge to the control of mosquito-borne diseases. In order to replenish our chemical “tool box”, mitigate insecticide resistance, and enhance our vector control capabilities, it is necessary to develop insecticides with novel mechanisms of action [6,7].

A physiological system in mosquitoes (and other insect pests/vectors) that has not been exploited to date for insecticide development is the renal excretory system. In the adult female mosquito, the only life stage that feeds on the blood of—and transmits pathogens to—vertebrate hosts, the Malpighian tubules play especially important roles in excreting (1) the excess water and ions that are absorbed into the extracellular fluid (hemolymph) after engorging on blood, and (2) the metabolic wastes that enter the hemolymph during the digestion and metabolism of blood (e.g., nitrogenous wastes). Thus, the Malpighian tubules offer a potential physiological target to exploit for developing insecticides that not only have novel mechanisms of action, but may also be highly selective to hematophagous adult females. The following pages highlight recent efforts by our group to understand the basic molecular mechanisms of Malpighian tubule function in adult female mosquitoes with the ultimate aim of developing novel mosquitocides.

## 2. Mosquito Malpighian Tubules

**Figure 1:**
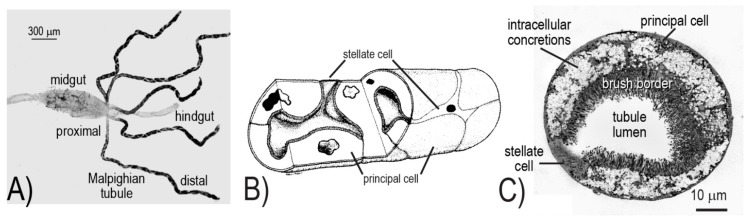
Anatomy and morphology of mosquito Malpighian tubules. (**A**) Isolated alimentary canal of an adult female mosquito (*Aedes aegypti*). The five Malpighian tubules connect to the alimentary canal at the junction of the midgut and hindgut. (**B**) Schematic of the distal end of a Malpighian tubule showing the arrangement of the principal and stellate cells. (**C**) Transverse section of a Malpighian tubule visualized via transmission electron microscopy. The principal cell contains a luminal brush border that is rich with mitochondria; the cell curls upon itself to form the tubule lumen. A stellate cell is also apparent. Panels A and C are from [8] with permission; panel B is from [11] with permission.

The morphology and physiology of mosquito Malpighian tubules have been the focus of recent comprehensive reviews [8,9,10]. In brief, the five Malpighian tubules together with the hindgut (i.e., ileum and rectum) form the renal excretory system of mosquitoes (Figure 1A). The Malpighian tubules produce urine via a “two-step” process of transepithelial fluid secretion. That is, ions (primarily Na^+^, K^+^ and Cl^−^) are actively transported by the tubule epithelial cells from the hemolymph to the tubule lumen, thereby generating an osmotic gradient for water to follow. The resulting urine is isosmotic to the hemolymph. The hindgut receives and modifies the urine (e.g., solute and/or water reabsorption), before excreting it from the mosquito via muscular contractions.

The cellular architecture of the Malpighian tubule epithelium is rather simple, consisting of only two different cell types: principal and stellate (Figure 1B,C). The principal cells are large, thick, mitochondrion-rich cells with an elaborate apical (luminal) brush border; they are fusiform in shape and fold upon themselves to form the tubule lumen (Figure 1B,C). On the other hand, stellate cells are small, thin, mitochondrion-poor cells with a highly in-folded basolateral (serosal) membrane; they possess a nucleated cell body with three to four arm-like projections that intercalate between principal cells, resulting in a “star-like” appearance (Figure 1B,C).

**Figure 2:**
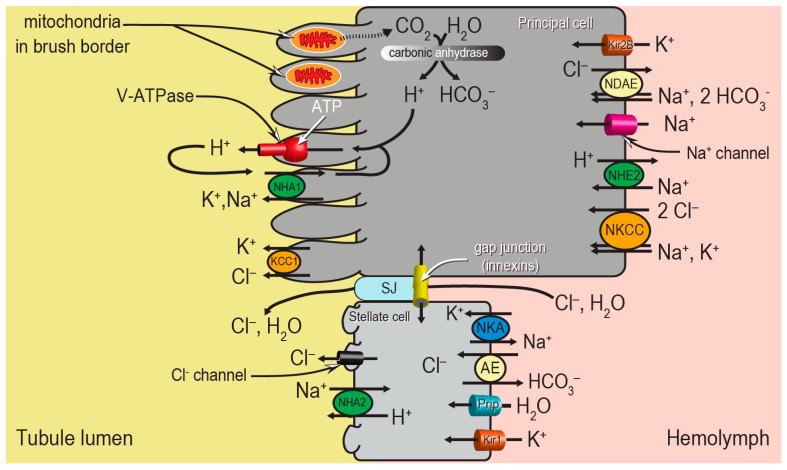
Model of transepithelial fluid secretion in mosquito Malpighian tubules. The transport modes of NHA1 and NHA2 are speculative. AE, Cl^−^/${\mathrm{HCO}}_{3}^{-}$ anion exchanger; ATP, adenosine triphosphate; KCC, K, Cl cotransporter; Kir, inward rectifier K^+^ channel; NDAE, Na^+^-driven anion exchanger; NHA, Na^+^/H^+^ antiporter; NHE, Na^+^/H^+^ exchanger; NKA, Na^+^-K^+^-ATPase; NKCC, Na^+^, K^+^, Cl^−^ cotransporter; Prip, *Pyroceoelia rufa* integral protein (aquaporin); SJ, septate junction. Modified from [12].

Despite the apparent morphological simplicity of the Malpighian tubule epithelium, the molecular mechanisms behind the “two-step” process of fluid secretion are complex, requiring the coordination of several ion transport mechanisms between the two cell types (Figure 2). Principal cells generate the primary electrochemical gradient that drives the secondary transport of ions and water from the hemolymph to the tubule lumen. In particular, a V-type H^+^-ATPase (V-ATPase) in the brush border pumps protons from the cytosol into the tubule lumen, thereby establishing an inside-negative voltage across both the apical and basolateral membranes of the epithelium. A Na^+^-K^+^-ATPase (NKA) on the basolateral membrane of stellate cells may play an accessory role to the V-ATPase. The negative membrane voltages drive ion uptake from the hemolymph across the basolateral membrane through (1) ion channels (e.g., Kir1, Kir2B, Na^+^ channel), and (2) potentially electrogenic transporters in the apical membranes (e.g., NHA1, NHA2). In addition to the conductive pathways, several electroneutral mechanisms for ion transport driven by chemical gradients are present in the basolateral and apical membranes, such as cation chloride cotransporters (e.g., Na^+^, K^+^, Cl^−^ cotransporter (NKCC), K^+^, Cl^−^ cotransporter (KCC1)), Na^+^/H^+^ exchangers (e.g., NHE2), and anion exchangers (e.g., Cl^−^/HCO_3_^−^ anion exchanger (AE), Na^+^-driven anion exchanger (NDAE)). The combined actions of the conductive and electroneutral ion transport mechanisms contribute to the movements of NaCl and KCl from the hemolymph into the tubule lumen, which provides the osmotic gradient for water to follow. This most likely occurs through stellate cells via aquaporin water channels (e.g., Prip) and/or the paracellular pathway via septate junctions (SJ) (Figure 2).

In theory, any of the above molecular mechanisms represent potential targets to exploit for disrupting urine production and thereby osmotic/ionic homeostasis in mosquitoes. For adult female mosquitoes, this disruption could be especially debilitating after engorging on blood, when they ingest over the equivalent of their body mass. In response to this engorgement, the Malpighian tubules mediate a pronounced diuresis that eliminates ~40% of the water and Na^+^, ~145% of the K^+^, and ~60% of the Cl^−^ from the ingested blood plasma within 2 h [13]. Moreover, limiting the capacity of Malpighian tubules to produce urine after a blood meal could dampen their capacity to excrete metabolic wastes associated with blood digestion (e.g., heme, nitrogenous wastes), which may lead to hemolymph poisoning and/or impaired egg production. It is with this vision in mind that motivated a collaborative effort by the Piermarini (The Ohio State University), Beyenbach (Cornell University), and Denton (Vanderbilt University) laboratories to identify K^+^ channels expressed in the Malpighian tubules of adult female mosquitoes and develop small-molecule inhibitors of these channels to disrupt the capacity of Malpighian tubules to produce urine, with the ultimate aim of disrupting K^+^ and fluid homeostasis in mosquitoes.

## 3. Targeting Potassium Channels in Mosquito Malpighian Tubules

Several lines of evidence strongly support the hypothesis that inward rectifier K^+^ (Kir) channels are major routes of K^+^ uptake across the basolateral membrane of mosquito Malpighian tubules, making them potential molecular targets for disrupting urine production. In isolated Malpighian tubules of the yellow fever mosquito *Aedes aegypti*, the Beyenbach laboratory has shown that barium, a canonical blocker of Kir channels, inhibits (1) over 50% of the transepithelial secretion of K^+^ and fluid, and (2) ~60% of the basolateral membrane conductance [14,15]. Moreover, the basolateral membrane of principal cells exhibits a cation permeability sequence (Tl^+^ >> K^+^ > Rb^+^ >> NH_4_^+^) consistent with the presence of Kir channels [16].

**Figure 3:**
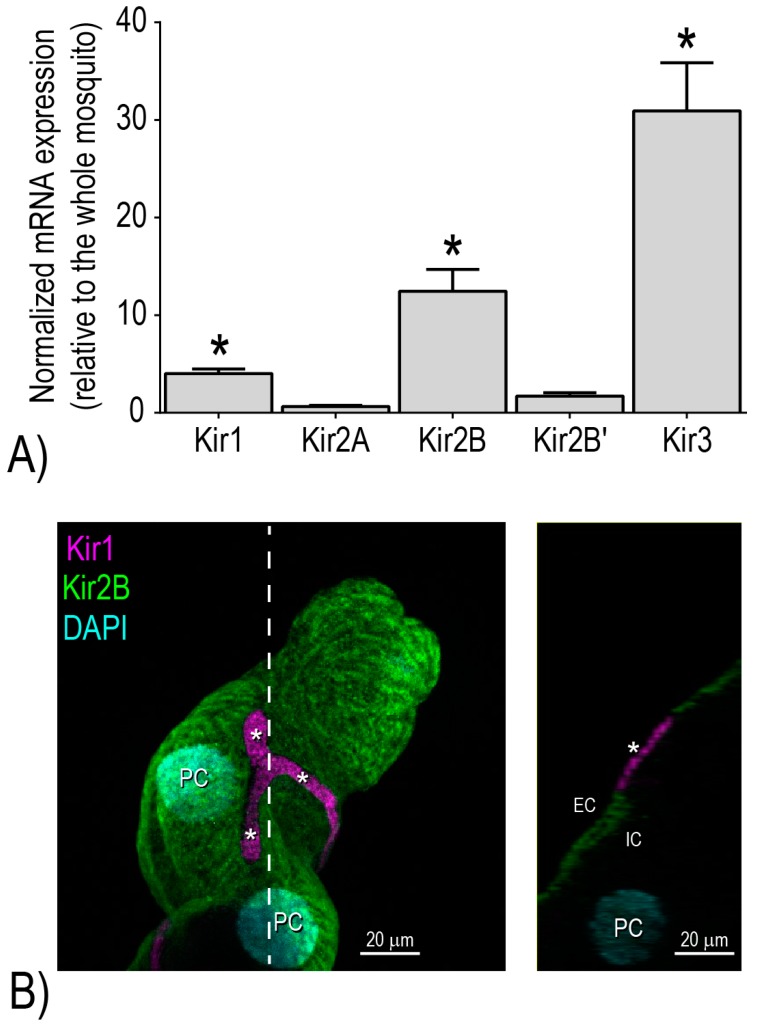
Molecular and immunochemical expression of Kir channel subunits in Malpighian tubules of adult female *Ae. aegypti*. (**A**) Relative mRNA expression of Kir subunits in Malpighian tubules normalized to expression in the whole mosquito. Values are means ± SEM. Asterisks indicate significantly enriched mRNA expression in the Malpighian tubules vs. the whole mosquito (*p* < 0.05). Data are from [22]. (**B**) Immunolocalization of *Ae*Kir1 (magenta) and *Ae*Kir2B (green) in Malpighian tubules. The left panel shows the cell-specific localization of *Ae*Kir1 to stellate cells (asterisks) and *Ae*Kir2B to principal cells. The right panel shows an optical section through the tubule in the plane indicated by the dashed line. Note the immunolabeling occurs along the basolateral region of each cell type. “EC” indicates extracellular space; “IC” indicates intracellular space; “PC” indicates nuclei of principal cells. Modified from [17] with permission.

Notably, we have shown that the Malpighian tubules of adult female *Ae. aegypti* are characterized by the enriched expression of three distinct mRNAs encoding Kir channel subunits: *Ae*Kir1, *Ae*Kir2B, and *Ae*Kir3 [16] (Figure 3A). When expressed heterologously in *Xenopus* oocytes, *Ae*Kir1 and *Ae*Kir2B each form functional channels mediating barium-inhibitable, inward-rectifying K^+^ currents [16]. Moreover, immunolabeling studies of Malpighian tubules localized *Ae*Kir1 and *Ae*Kir2B to the basolateral membranes of stellate and principal cells, respectively [17] (Figure 3B). On the other hand, *Ae*Kir3 does not form functional K^+^ channels in the plasma membrane when heterologously-expressed in *Xenopus* oocytes [16] or HEK-293 cells (Denton, personal observation), and its immunoreactivity localizes to intracellular compartments of principal and stellate cells in Malpighian tubules [17]. Thus, *Ae*Kir1 and *Ae*Kir2B appear to be the best candidates for mediating the barium-sensitive transepithelial transport of K^+^ and fluid in mosquito Malpighian tubules.

**Figure 4:**
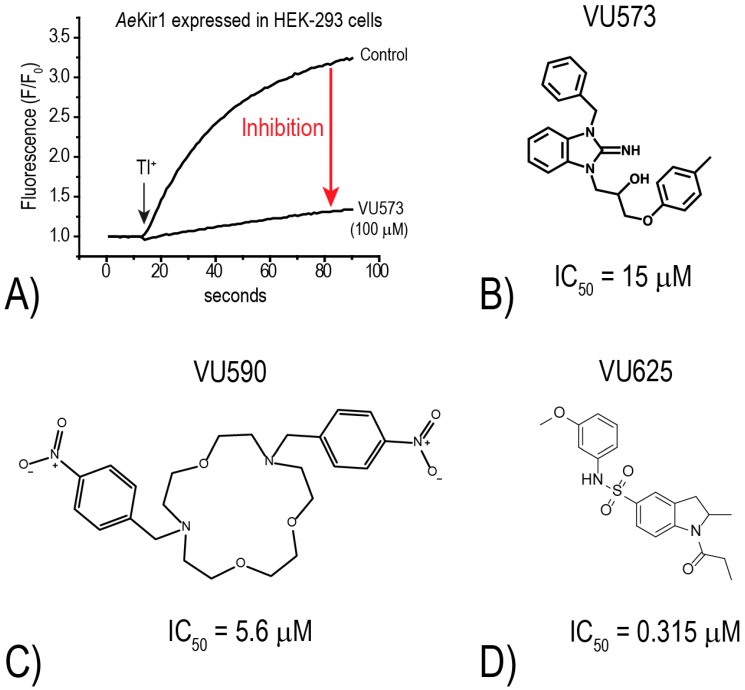
Discovery of small molecule inhibitors of *Ae*Kir1. (**A**) Tl^+^-flux assay showing the rapid increase of fluorescence (F/F_0_) in HEK-293 cells expressing *Ae*Kir1 upon addition of Tl^+^ (black arrow) to the extracellular bath (Control). If the cells are incubated with VU573 (100 μM) prior to addition of Tl^+^, the increase of fluorescence is nominal, suggesting inhibition of *Ae*Kir1. Modified from [18]. (**B**–**D**) Structures of small molecule inhibitors of *Ae*Kir1 discovered by the Denton laboratory. The respective potencies of the compounds (IC_50_) in the Tl^+^ flux assays are indicated. Data are from [18,19,20].

To further explore this hypothesis, the Denton laboratory developed a thallium (Tl^+^) flux assay for *Ae*Kir1 (expressed heterologously in HEK-293 cells) to allow for the high-throughput discovery of novel small-molecule inhibitors of mosquito Kir channels [18]. The assay utilizes an intracellular, Tl^+^-sensitive fluorescent dye and follows the principle that Kir channels mediate the uptake of Tl^+^ into cells. Thus, upon the addition of Tl^+^ to the extracellular bath of cells expressing functional Kir channels, intracellular fluorescence increases as Tl^+^ moves through the Kir channel pore (e.g., “Control” in Figure 4A). In the presence of a small molecule inhibitor, the rate of fluorescence increase is reduced (e.g., “VU573” in Figure 4A). Using this Tl^+^-flux assay, we have screened over 75,000 small molecules from the Vanderbilt Institute of Chemical Biology library against mosquito Kir1 and identified over 300 potential inhibitors. To date, we have focused most of our attention on three small molecule inhibitors of *Ae*Kir1: VU573, VU590, and VU625 (Figure 4B–D). Intriguingly, each of these *Ae*Kir1 inhibitors has a unique effect on *Ae*Kir2B (expressed heterologously in *Xenopus* oocytes). That is, the K^+^ currents mediated by *Ae*Kir2B are (1) stimulated by VU573, (2) not affected by VU590, and (3) are inhibited by VU625 [19,20].

**Figure 5:**
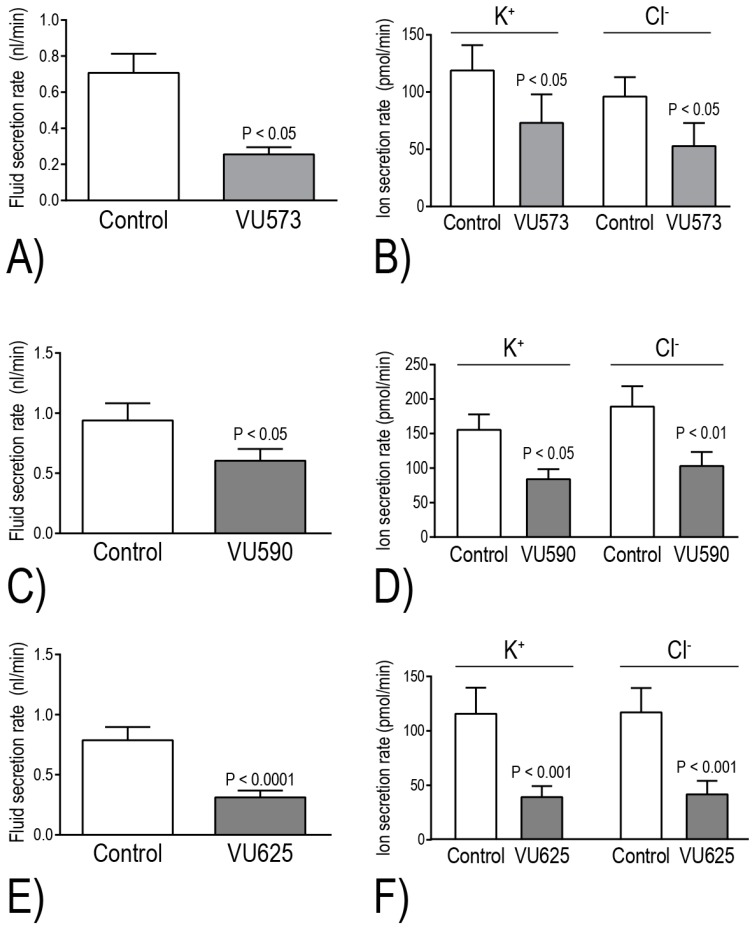
Effects of small molecule inhibitors of *Ae*Kir1 (10 μM) on fluid (**A**, **C**, **E**) and KCl (**B**, **D**, **F**) secretion in isolated Malpighian tubules of *Ae. aegypti* as determined via the Ramsay assay. The experiments were performed on “sister” tubules isolated from the same mosquitoes. In the control tubules, 0.05% dimethyl sulfoxide (DMSO, the vehicle for small molecules) was added to the peritubular bath instead of the indicated small molecule. The secretion rates shown were measured 2 h after the addition of DMSO or the small molecule. Values are means ± SEM. Data are from [17,21].

The Beyenbach laboratory has evaluated the effects of VU573, VU590, and VU625 on isolated Malpighian tubule function in vitro via the Ramsay assay of fluid secretion. Within 2 h of adding one of these molecules to the peritubular bath, the secretion of fluid and KCl is reduced by ~40%–60%, consistent with the inhibition of basolateral Kir channels [17,18,21] (Figure 5). Taking into consideration the aforementioned differential pharmacological effects of the small molecules on *Ae*Kir1 vs. *Ae*Kir2B, it was possible to dissect the relative contributions of each Kir channel to transepithelial K^+^ secretion in isolated Malpighian tubules. Notably, VU590 and VU625 decrease transepithelial K^+^ secretion by 46% and 66%, respectively [17] (Figure 5D,F). These data indicate that *Ae*Kir1 in stellate cells and *Ae*Kir2B in principal cells respectively contribute to ~50% and 20% of the transepithelial K^+^ transport. Thus, the inhibition of at least *Ae*Kir1 can potentially have major impacts on mosquito urinary K^+^ excretion and thereby hemolymph K^+^ homeostasis.

**Figure 6:**
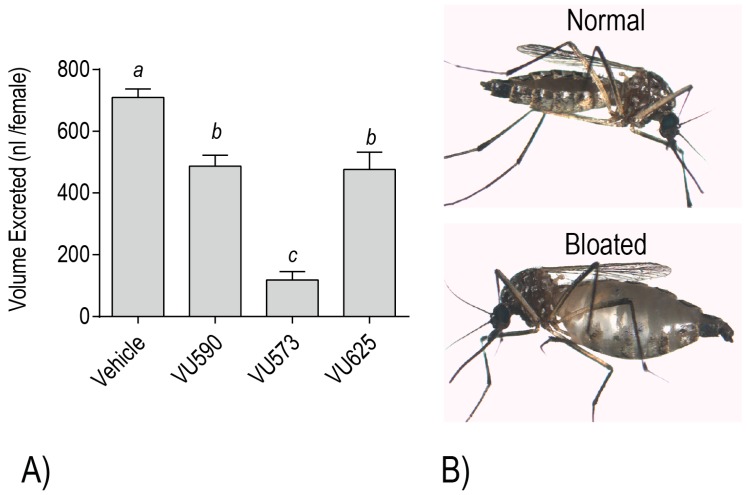
(**A**) Effects of small molecule inhibitors of *Ae*Kir1 on the diuretic capacity of adult female mosquitoes (*Ae. aegypti*). Small molecules (690 pmol per mosquito) were delivered to the hemolymph along with 900 nL of phosphate-buffered saline (PBS) via microinjection to stimulate diuresis. The amount of urine excreted was measured 60 min after injection. The vehicle consisted of PBS containing 1.15% DMSO, 0.077% β-cyclodextran, and 0.008% Solutol. Values are means ± SEM. Lowercase letters indicate statistical categorization of the means as determined by a one-way ANOVA with a Newman-Keuls posttest (*p* < 0.05). Data are from [19,20,21]. (**B**) Adult females with normal and bloated abdomens 24 h after hemolymph injection of the vehicle or VU573, respectively. Similar results were observed after injection with VU590 [20]. Images are from [18].

To further test this possibility, we evaluated the effects of VU573, VU590, and VU625 on mosquito behavior/survival and excretory function. Notably, injection of each small molecule into the hemolymph of adult female *Ae. aegypti* elicits dose-dependent toxic effects within 24 h, manifested as death or a loss of flight [18,19,20]. Each of the molecules also reduces the whole mosquito capacity for diuresis, consistent with an inhibitory effect on the renal excretory system [18,19,20,21] (Figure 6A). Moreover, mosquitoes treated with VU573 are more susceptible to a hemolymph load of K^+^, which is likely a consequence of a decreased capacity for renal K^+^ excretion [18,21]. Among the more dramatic renal effects that manifest in adult female mosquitoes after hemolymph injection of VU573 or VU590 is extreme abdominal swelling, presumably due to extracellular fluid retention associated with impaired renal function [18,20] (Figure 6B). In some cases, we have observed that the swelling causes a physical rupturing of the abdominal wall (Piermarini, unpublished observations).

To confirm that the above effects on Malpighian tubules and mosquitoes were specific to the inhibition of *Ae*Kir1, we developed so-called “inactive” analogs of each molecule that do not inhibit the activity of *Ae*Kir1 in vitro. Importantly, the inactive analogs neither inhibit fluid/K^+^ secretion in isolated Malpighian tubules nor exhibit toxic effects on adult female mosquitoes when injected into the hemolymph [17,18,19,20,21]. Taken together, the above in vitro and in vivo results indicate that the inhibition of *Ae*Kir1 disrupts K^+^ and fluid secretion at the level of the Malpighian tubules, leading to disruptions of hemolymph K^+^ and fluid homeostasis at the level of the whole mosquito. In other words, small molecule inhibitors of *Ae*Kir1 elicit renal failure in mosquitoes, suggesting that they have potential for development into mosquitocides with novel mechanisms of action.

**Figure 7:**
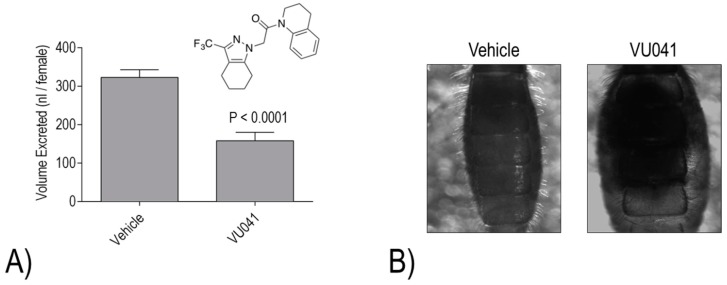
(**A**) Effects of VU041 (structure shown) on the diuretic capacity of adult female mosquitoes (*Ae. aegypti*). The mosquitoes were topically-treated with a sub-lethal dose of VU041 (1.6 μg/mg mosquito) 2 h prior to injection of their hemolymph with 900 nL of PBS. Mosquitoes treated with the vehicle (50% acetone/50% ethanol) served as controls. The amount of urine excreted within one hour of injection was measured. Values are means ± SEM. (**B**) Representative images of the abdomens of adult female *An. gambiae* 24 h after a blood meal. The mosquitoes were treated with a sub-lethal dose VU041 (1.0 μg/mg mosquito) within 30 min after engorgement. Mosquitoes treated with the vehicle (95% ethanol) served as controls. Data and images are from [23].

Despite the promising activities of VU573, VU590, and VU625, they each share one common limitation for mosquitocide development: they are unable to elicit toxic effects when applied topically to the cuticle. Thus, our most recent effort has focused on a small molecule inhibitor of *Ae*Kir1 (i.e., VU041) with a high calculated permeability coefficient (cLogP), which makes a molecule more likely to penetrate the cuticle of insects [23]. VU041 was discovered by the Denton laboratory in a high-throughput screen for inhibitors of the Kir1 ortholog of *Anopheles gambiae* (*An*Kir1) [23]; it is equally effective against *Ae*Kir1 [23]. Notably, VU041 is toxic to adult female *Ae. aegypti* and *An. gambiae* within 24 h of topical application to the cuticle [23]. Moreover, VU041 exhibits similar efficacy against insecticide-susceptible and -resistant strains of both mosquito species [23]. We have confirmed that topical application of VU041 inhibits the diuretic capacity of *Ae. aegypti*, consistent with an effect on the Malpighian tubules [23] (Figure 7A). Furthermore, in *An. gambiae*, topical treatment with VU041 leads to apparent fluid retention in the abdomen 24 h after a blood meal [23] (Figure 7B). These effects on the renal system likely contribute to impaired blood meal processing/metabolism as indicated by the decreased fecundity of both species if treated with VU041 after engorgement [23]. Thus, VU041 provides a promising chemical scaffold for developing a next-generation mosquitocide with a novel mechanism of action (i.e., renal failure) that can potentially be used to mitigate resistance of mosquitoes to conventional insecticides.

## 4. Finding New Targets: Transcriptomic Insights into Malpighian Tubule Function in Blood Fed Mosquitoes

As described above, the Malpighian tubules of mosquitoes play a key role in mediating the post-prandial diuresis, which alleviates the acute challenges to hemolymph salt and water balance after engorging on blood. However, once the post-prandial diuresis subsides (~1–2 h post-blood meal), the potential roles of the Malpighian tubules in detoxifying and excreting metabolites generated during blood meal digestion, such as heme and nitrogenous wastes (e.g., NH_3_), have not been widely investigated. Thus, we initiated transcriptomic studies on the Malpighian tubules of the Asian tiger mosquito, *Aedes albopictus*, before and after a blood meal to generate insights.

**Figure 8:**
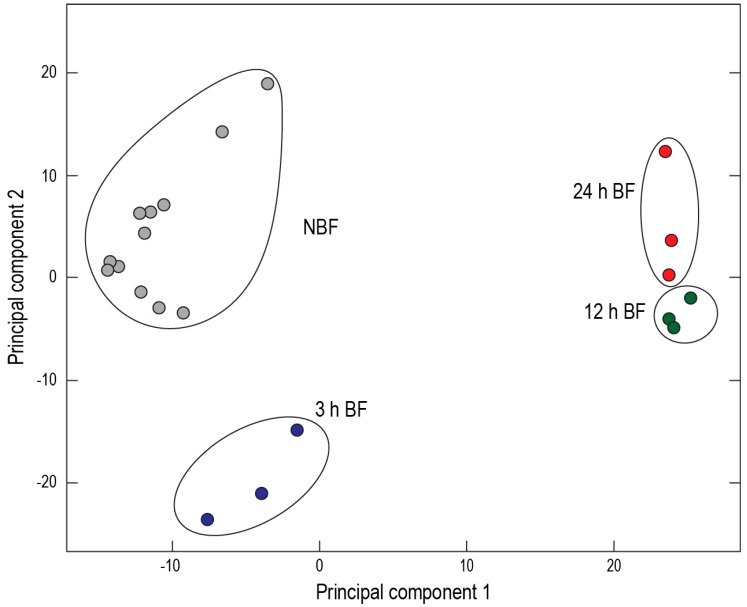
Principal component analysis of transcript expression in Malpighian tubules from non-blood fed (NBF) and blood-fed (BF) mosquitoes (*Ae. albopictus*). Each circle represents a sequenced cDNA library (via RNA-Seq). Note that the libraries cluster spatially by treatment and time. Reprinted from [12].

In collaboration with Dr. Bryan Cassone (Brandon University), we used RNA-Seq to characterize and quantify global transcript expression in Malpighian tubules isolated from *Ae. albopictus* at 3 h, 12 h, and 24 h post-blood meal (all compared to control Malpighian tubules from non-blood fed mosquitoes). Within 24 h post-blood meal, over 80% of the ingested protein is digested [24]; thus, it is a period of intense metabolic activity. The changes to transcript expression in the Malpighian tubules after blood feeding were dramatic: over 3200 transcripts (i.e., ~40% of the transcriptome) were differentially-expressed at each time point relative to non-blood fed controls [12,25]. Moreover, the changes to transcript expression in the Malpighian tubules after blood feeding were dynamic: there were roughly equal numbers of up- and down-regulated transcripts at each time point [12,25]. Furthermore, a two-dimensional principal component analysis of transcript expression demonstrated spatial clustering of the sequenced cDNA libraries by treatment and time point (Figure 8). These findings suggest that the Malpighian tubules of *Ae. albopictus* are in the midst of a functional transition after a blood meal.

**Figure 9:**
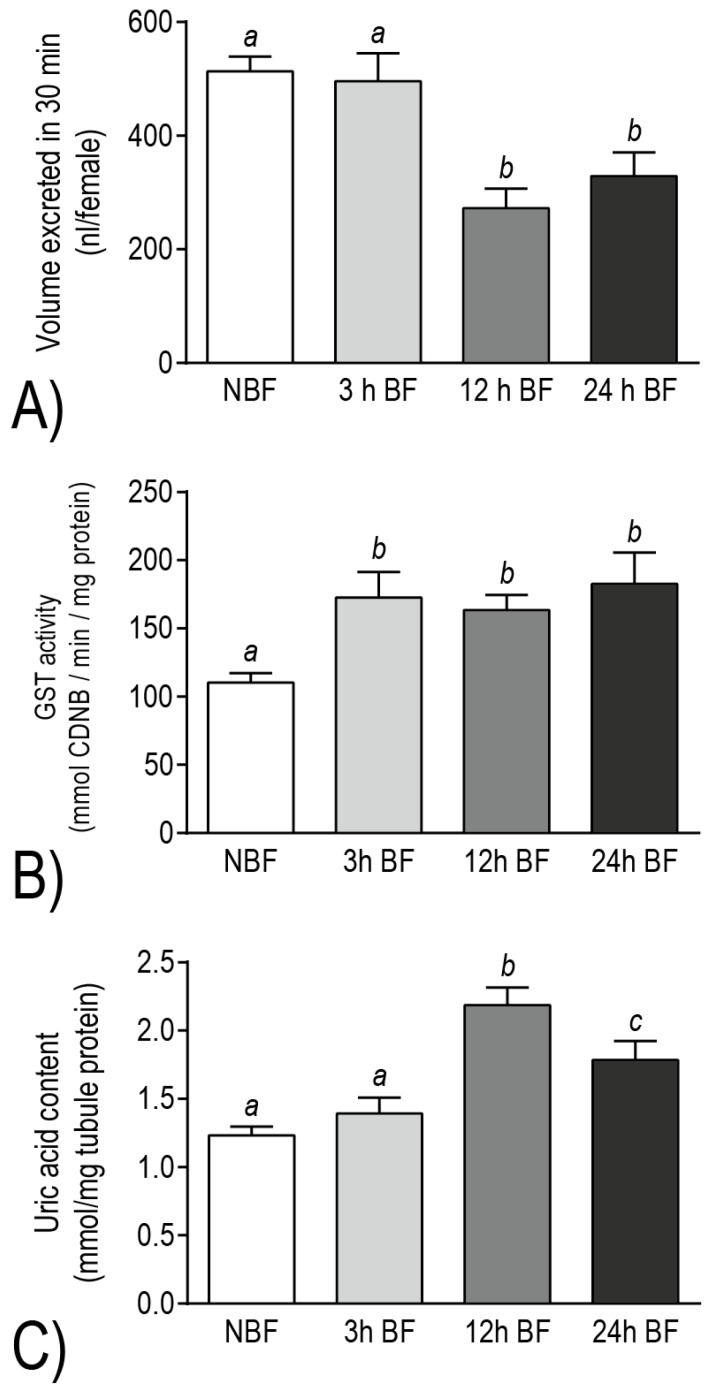
Physiological and biochemical evidence for a functional transition in Malpighian tubules of adult female *Ae. albopictus* within 24 h after a blood meal. (**A**) Effect of a blood meal on the whole mosquito diuretic capacity. The hemolymph of mosquitoes was microinjected with 900 nL of PBS and the amount of urine excreted was measured 30 min after injection. (**B**) Effect of a blood meal on the biochemical activity of glutathione *S*-transferase (GST) in Malpighian tubules. The assay measured the GST-mediated conjugation of reduced glutathione to 1-chloro-2,4-dinitrobenzene (CDNB), a colorimetric substrate. (**C**) Effect of a blood meal on the amount of uric acid in Malpighian tubules. In all panels: NBF = non-blood fed mosquitoes and BF = blood fed mosquitoes; values are means ± SEM; and lowercase letters indicate statistical categorization of the means as determined by a one-way ANOVA with a Newman-Keuls posttest (*p* < 0.05). Modified from [12].

To further explore this notion, we conducted a Database for Annotation, Visualization and Integrated Discovery (DAVID) functional cluster analysis [26,27] on the differentially-expressed transcripts and performed functional assays on mosquitoes and Malpighian tubules [12,25]. Among the transcripts that were down-regulated 3–24 h after a blood meal, we identified DAVID molecular pathways (e.g., oxidative phosphorylation, ATPase) and gene families (e.g., V-ATPase subunits, aquaporins) associated with active transepithelial fluid secretion [12,25]. Consistent with these transcript changes, adult female mosquitoes exhibited a decreased capacity for diuresis at 12 h and 24 h post-blood meal relative to non-blood fed females [12] (Figure 9A). On the other hand, among the transcripts that were up-regulated after a blood meal, we identified DAVID molecular pathways (e.g., thioredoxin, ATP-binding cassette transporter, cytochrome P450, tryptophan oxidation) and gene families (e.g., glutathione *S*-transferases, glutathione peroxidases, glutaredoxins, xanthine dehydrogenases) associated with redox homeostasis and metabolite detoxification [12,25]. Consistent with these transcript changes, Malpighian tubules exhibited biochemical increases of (1) glutathione *S*-transferase (GST) activity at 3–24 h post-blood meal and (2) uric acid at 12–24 h post-blood meal, relative to the Malpighian tubules of non-blood fed controls [12] (Figure 9B,C). Thus, the molecular and physiological/biochemical results both indicate that the Malpighian tubules of *Ae. albopictus* undergo a functional transition after a blood meal, wherein molecular resources appear to be diverted away from mechanisms of diuresis and are reinvested into mechanisms associated with redox homeostasis and detoxification of blood-meal metabolites.

**Figure 10:**
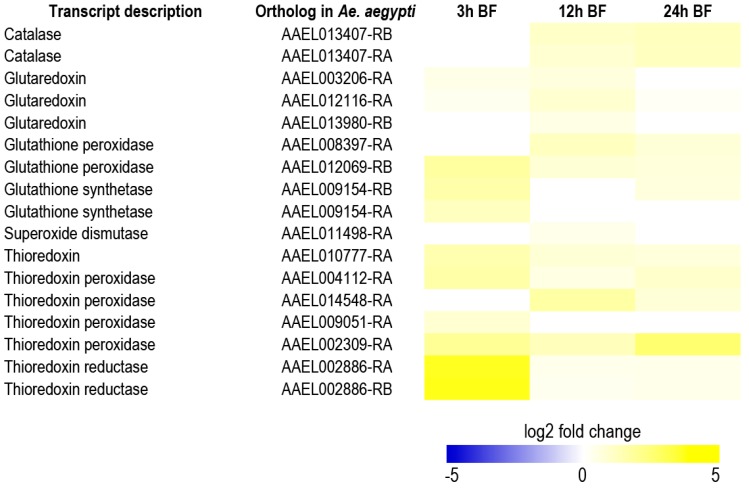
Heat map of transcripts encoding antioxidant enzymes that are significantly up-regulated in the Malpighian tubules of blood fed (BF) adult female *Ae. albopictus*. The log2 fold changes in transcript expression are indicated by the provided color scale and are relative to Malpighian tubules of non-blood fed mosquitoes. Data are from [12].

**Figure 11:**
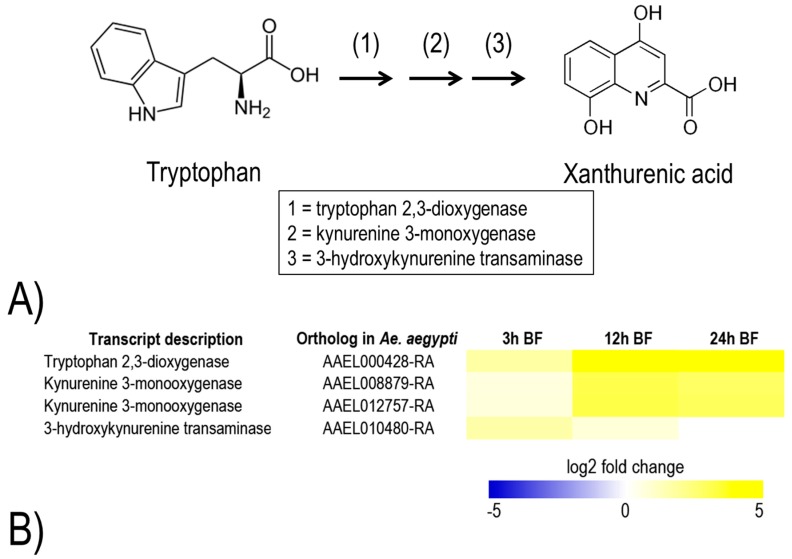
(**A**) Metabolic pathway for synthesis of xanthurenic acid from tryptophan. (**B**) Heat map of transcripts encoding enzymes associated with xanthurenic acid synthesis that are significantly up-regulated in the Malpighian tubules of blood fed (BF) adult female *Ae. albopictus*. The log2 fold changes in transcript expression are indicated by the provided color scale and are relative to Malpighian tubules of non-blood fed mosquitoes. Data are from [12].

Although additional efforts are required to validate that the up-regulated metabolic pathways and transcripts in the Malpighian tubules are vital for mosquito survival and/or fecundity after a blood meal, they may offer unexploited molecular targets for developing insecticides with novel modes of action. For example, several transcripts that encode putative antioxidant enzymes are up-regulated in Malpighian tubules within 24 h post-blood meal (Figure 10). Notably, in some cases, the transcript up-regulation is dramatic, such as two thioredoxin reductases at 3 h (log2 fold change = 3.7) and a thioredoxin peroxidase (AAEL002309-RA) at 24 h (log2 fold change = 2.6). In addition, a metabolic pathway associated with the production of xanthurenic acid, a chelator of heme and iron [28], from tryptophan is up-regulated within 24 h (Figure 11). In particular, transcripts predicted to encode tryptophan 2,3-dioxygenase and kynurenine 3-monoxygenase are highly up-regulated at 12 h and 24 h (log2 fold change = 2.8 to 5.4). Notably, in the Malpighian tubules of *An. gambiae*, a transcript for kynurenine 3-monoxygenase was also highly enriched at 3 h post-blood meal [29]. These findings suggest that the Malpighian tubules contribute to antioxidant production and heme detoxification in mosquitoes during blood meal processing. An alternate, but not mutually-exclusive, explanation is that the Malpighian tubules are highly-susceptible to heme-induced, oxidative tissue damage, and thereby require an up-regulation of antioxidant and heme-chelating mechanisms after a blood meal to maintain normal function. Thus, inhibiting one or more of the above mechanisms with small molecules may disrupt the renal contributions to hemolymph redox homeostasis and/or Malpighian tubule function in general after blood feeding, which may lead to premature death and/or reduced fecundity.

High-throughput assays for discovering small-molecule inhibitors of mammalian thioredoxin reductase, tryptophan 2,3-dioxygenase, and kynurenine 3-monoxygenase, which are human drug targets, have already been developed [30,31,32]. Moreover, inhibitors of mosquito tryptophan 2,3-dioxygenase and 3-hydroxykynurenrine transaminase have already been discovered, some of which exhibit larvicidal activity [33,34]. Thus, pursuing a small-molecule approach, parallel to the one we have used for Kir channels, may be warranted for identifying potential mosquitocides that disrupt the antioxidant and xanthurenic acid-synthesis enzymes up-regulated in the Malpighian tubules after a blood meal.

## 5. Conclusions

Resistance of mosquitoes to conventional insecticides targeting the nervous system limits our capacity to control the spread of emerging and re-emerging mosquito-borne diseases. Thus, development of insecticides with novel modes of action is critical to enhance vector control capabilities. The work highlighted in the present review demonstrates that the Malpighian tubules offer a new physiological target to exploit for insecticide development. In particular, inhibiting Kir channels of adult female mosquitoes with small molecules disrupts the renal regulation of hemolymph K^+^ and fluid homeostasis, leading to toxicity via a completely novel mode of action from conventional insecticides. Transcriptomic studies on Malpighian tubules of blood fed mosquitoes reveal an up-regulation of numerous molecular mechanisms associated with the detoxification and excretion of harmful metabolites, such as heme. Targeting one or more of these renal mechanisms with small molecules could lead to the development of insecticides with novel modes of action that shorten the mosquito life span and/or decrease fecundity by disrupting blood meal processing. Future studies validating the importance of specific up-regulated mechanisms in the survival and fecundity of blood-fed mosquitoes will facilitate the prioritization of molecular targets for insecticide development.
